# SLEEP ARCHITECTURE IN OLDER ADULT INTENSIVE CARE UNIT SURVIVORS

**DOI:** 10.1093/geroni/igae098.3525

**Published:** 2024-12-31

**Authors:** Priscilla Carmiol-Rodriguez, Maya Elias

**Affiliations:** University of Washington, Washington, United States; University of Washington, Seattle, Washington, United States

## Abstract

Sleep is highly fragmented in older adults recovering from critical illness, despite discharge out of an intensive care unit (ICU). The aim of this study was to measure nighttime sleep architecture in older ICU survivors after ICU discharge to a post-ICU inpatient unit. The sample consisted of 13 older ICU survivors who were functionally independent prior to hospitalization, mechanically ventilated in ICU, and enrolled within 24-48 hours after ICU discharge at an academic medical center. Sleep architecture was measured via a portable polysomnography sleep monitoring device (Sleep Profiler) between 10:00 PM-6:00 AM over two consecutive nights. Data were analyzed using descriptive statistics, and the one-sample Wilcoxon signed rank test evaluated the differences between the time spent in each sleep stage in our sample of older ICU survivors versus the established norms for healthy older adults. Among our sample, participants were awake approximately 53.4% (interquartile range [IQR]: 45.0) of nighttime hours. The median percentages of the sleep stages were: stage N1 = 2.1% (IQR: 6.65); stage N2 = 26.35% (IQR: 41.63); stage N3 = 0.8% (IQR: 6.33); and stage REM = 0.15% (IQR: 7.6). Results indicated that the median percentages of the sleep stages N1, N2, N3, and REM were significantly lower than the expected reference values for each sleep parameter (p < 0.05). Therefore, older ICU survivors may experience insufficient slow wave sleep and REM sleep, which are vital for recovery from critical illness. Further interdisciplinary research is needed to optimize sleep in the post-ICU inpatient setting.

